# Impact of severe mental illness on healthcare use and health outcomes for people with type 2 diabetes: a longitudinal observational study in England

**DOI:** 10.3399/BJGP.2020.0884

**Published:** 2021-06-29

**Authors:** Lu Han, Tim Doran, Richard Ian Gregory Holt, Catherine Hewitt, Rowena Jacobs, Stephanie Louise Prady, Sarah Louise Alderson, David Shiers, Han-I Wang, Sue Bellass, Simon Gilbody, Charlotte Emma Wray Kitchen, Jennie Lister, Johanna Taylor, Najma Siddiqi

**Affiliations:** Department of Health Services Research and Policy, London School of Hygiene and Tropical Medicine, London.; Department of Health Sciences, University of York, York.; Faculty of Medicine, University of Southampton, Southampton.; Department of Health Sciences, University of York, York.; Centre for Health Economics, University of York, York.; Department of Health Sciences, University of York, York.; Leeds Institute for Health Sciences, University of Leeds, Leeds.; University of Manchester, Manchester; honorary research consultant, Psychosis Research Unit, Greater Manchester Mental Health NHS Foundation Trust, Manchester; honorary senior research fellow, Keele University, Keele.; Department of Health Sciences, University of York, York.; Leeds Institute for Health Sciences, University of Leeds, Leeds.; Department of Health Sciences, University of York, York.; Department of Health Sciences, University of York, York.; Department of Health Sciences, University of York, York.; Department of Health Sciences, University of York, York.; Department of Health Sciences, University of York, York.

**Keywords:** cardiovascular diseases, diabetes, mortality, primary care, severe mental illness

## Abstract

**Background:**

People with severe mental illnesses (SMIs) have reduced life expectancy compared with the general population. Diabetes is a contributor to this disparity, with higher prevalence and poorer outcomes in people with SMI.

**Aim:**

To determine the impact of SMI on healthcare processes and outcomes for people with type 2 diabetes (T2DM).

**Design and setting:**

Retrospective, observational, matched, nested, case–control study conducted in England using patient records from the Clinical Practice Research Datalink, linked to Hospital Episode Statistics.

**Method:**

A range of healthcare processes (primary care consultations, physical health checks, and metabolic measurements) and outcomes (prevalence and hospitalisation for cardiovascular disease [CVD], and mortality risk) were compared for 2192 people with SMI and T2DM (cases) with 7773 people who had diabetes alone (controls). Sociodemographics, comorbidity, and medication prescription were covariates in regression models.

**Results:**

When compared with results for participants with T2DM only, SMI was associated with increased risk of all-cause mortality (hazard ratio [HR] 1.919, 95% confidence interval [CI] = 1.602 to 2.300) and CVD-specific mortality (HR 2.242, 95% CI = 1.547 to 3.250), higher primary care physician consultation rates (incidence rate ratio [IRR] 1.149, 95% CI = 1.111 to 1.188), more-frequent checks of blood pressure (IRR 1.024, 95% CI = 1.003 to 1.046) and cholesterol (IRR 1.038, 95% CI = 1.019 to 1.058), lower prevalence of angina (odds ratio 0.671, 95% CI = 0.450 to 1.001), more emergency admissions for angina (IRR 1.532, 95% CI = 1.069 to 2.195), and fewer elective admissions for ischaemic heart disease (IRR 0.682, 95% CI = 0.508 to 0.915).

**Conclusion:**

Monitoring of metabolic measurements was comparable for people with T2DM who did, and did not, have SMI. Increased mortality rates observed in people with SMI may be attributable to underdiagnosis of CVD and delays in treatment.

## INTRODUCTION

The average life expectancy for people with severe mental illnesses (SMIs), such as schizophrenia or bipolar disorder, is 7–24 years lower than that for the general population.^1^ Higher prevalence of noncommunicable diseases in people with SMI is a key contributor to this disparity,^2^^–^5 partly driven by socioeconomic disadvantage, health-risk behaviours,^6^^,^^7^ and side-effects of medications.^8^^–^10 Co-existing SMI and comorbid conditions may interact, resulting in poorer outcomes for both, and access to health care for physical problems may also be more problematic for people with SMI compared to people without SMI.^11^

In the UK, the prevalence of type 2 diabetes (T2DM) is twice as high in people with SMI compared with the general population;^12^ these individuals also have an increased incidence of acute metabolic emergencies and diabetes complications.^13^^–^14 National guidelines, therefore, recommend regular screening for diabetes in people with SMI, with the aim of achieving the same standards of care as for the general population.^15^^–^19 The UK’s primary care pay- for-performance programme, the Quality and Outcomes Framework (QOF), has included quality targets for both diabetes and SMI since 2004.^20^

Overall, recorded quality of care for diabetes has improved substantially following the introduction of national quality-improvement initiatives.^21^ However, evidence is lacking on the appropriateness and effectiveness of universal quality targets in subgroups of people with diabetes, including those with SMI, and little is known about how SMI and other risk factors combine to affect diabetes outcomes. A linked healthcare dataset was used, therefore, to investigate, in people with diabetes, the impact of SMI on healthcare processes and diabetes outcomes, including the use of routine primary care services, metabolic monitoring, diagnosis and hospitalisation rates for cardiovascular disease (CVD), and the risk of mortality. The aim of this study was to identify potential elements in the care pathway that might be associated with increased risk of mortality in people with SMI.

**Table:** How this fits in

People with severe mental illnesses (SMIs) have poorer physical health and a life expectancy that is lower than the general population. Diabetes contributes significantly to this health inequality. National UK guidelines have recommended regular screening for diabetes in people with SMI and monitoring of metabolic risk factors in people with diabetes. This study, conducted in England, provides new evidence that the monitoring of diabetes and metabolic control is no worse for people with SMI and diabetes, compared with that for people with diabetes alone. However, people with SMI are underdiagnosed for cardiovascular disease (CVD) in primary care and, consequently, have poorer access to specialist and elective hospital care; this may explain the elevated risk of mortality due to CVD in this population.

## METHOD

### Data sources and participants

The dataset was extracted from Clinical Practice Research Datalink (CPRD) GOLD, which includes patient information on symptoms and diagnoses, referrals to specialists and secondary care settings, prescriptions issued in primary care, diagnostic testing, biometric data, and other types of care as routinely provided in primary care. Patient characteristics are broadly representative of the general UK population in terms of age, sex, and ethnicity.^22^ Individual patient data were electronically linked to external data sources, including Hospital Episode Statistics for hospital admissions, Office for National Statistics for death records, and the Index of Multiple Deprivation (IMD) for area deprivation.^23^^–^25

A matched, nested, case–control design was used. Cases comprised people with comorbid SMI and T2DM; people with T2DM but no SMI were identified as matched controls based on age, sex, and primary care practice, with a maximum ratio of 4:1. SMI was defined as the presence of at least one diagnostic record entry for schizophrenia, schizoaffective disorder, bipolar disorder, depression, or other affective disorder with psychosis in either primary care or hospital-admission data. Diabetes was classified as the presence of diagnostic codes for T2DM in primary care or hospital-admission data. Cases and controls were included if the patient:

- had health records that were up to research standard and eligible for relevant linkages;
- was registered with a participating primary care practice in England in the study period (1 April 2000–31 March 2016);
- was aged ≥18 years when diagnosed with either T2DM or SMI;
- had a continuous medical record; and
- could be nested within a matched case–control cluster.

Individual follow-up periods started on the latter date of either the T2DM diagnosis or the beginning of data that were up to research standards plus 15 months, to ensure there was a large window for observing baseline participant characteristics. Follow-up ended on the earlier date of 31 March 2016 or the end of data that were up to research standards (see Supplementary Figure S1).

### Variables

The exposure variable was SMI diagnosis. Outcome variables were primary care consultations, completion of physical health checks, metabolic measurements, diagnosis and hospitalisation for CVD, and risk of all-cause and CVD-specific mortality.

Primary care consultation rates were expressed as the average number of face-to-face consultations per year with practice-based health professionals in the follow-up period. The average number of health checks per year was calculated as recorded checks on blood pressure (BP), serum cholesterol, glycated haemoglobin (HbA1c), and body mass index (BMI) as incentivised under the QOF for people with T2DM. Metabolic measurements were expressed by the average levels of BP, serum cholesterol, and HbA1c in the study period.

CVD was identified as the presence of diagnostic codes recorded in primary care data during the follow-up period, with:

- separate indicators for:
  − angina;
  − myocardial infarction (MI), including acute coronary syndrome;
  − stroke;
  − chronic ischaemic heart disease (IHD); and
- a combined indicator for macrovascular complications (MI, stroke, and peripheral vascular disease [PVD]).

Hospital admissions for CVD were measured as the average number of admissions per year in the follow-up period, separated by emergency and elective admissions, as well as by diagnosis groups, including angina, MI, chronic IHD, and stroke.

Adjustments were made for age, ethnicity, and area deprivation — the latter was obtained by linking participants’ residential postcodes to the 2010 English IMD at the Lower Layer Super Output Area level, and dividing into quintiles. Baseline comorbidities were measured by the diagnosis of CVD, hypertension, dementia, learning disability, and the number of Charlson Comorbidity Index (CCI) comorbidities (excluding diabetes and diabetes complications) prior to follow-up. Baseline medication use was determined through documentation of at least one prescription for antidepressants and antipsychotics (first and second generations), or antihypertensive, antidiabetes, or lipid-lowering medications issued in the 15-month window prior to follow-up. Baseline smoking status (as a health-risk behaviour) and biometric measures (BMI, BP, serum cholesterol, and HbA1c) were constructed using the most-recent records extracted from the 15-month window.

### Statistical methods

A case–control cluster entered the analysis only after the patient constituting the ‘case’ was diagnosed with SMI, if T2DM was diagnosed first. ‘Cases’ who received a diagnosis of SMI before T2DM were not affected by this. Within estimators were applied in the regressions to examine the variations in outcomes among matched individuals only. Conditional logistic regression models, Poisson, or negative binomial models and stratified Cox proportional hazard models were applied, depending on the type of outcome variables.

SMI status was treated as time dependent in survival analyses, and the proportional hazard assumption was tested by the inclusion of interaction effects between explanatory variables and time — interaction terms with statistically significant coefficients were retained in the final models. Goodness of fit was assessed by the C-statistic, the Akaike information criterion, and the Bayesian information criterion, as appropriate. Due to the extent of baseline characteristic data that were missing, each participant’s family history of diabetes, smoking status, and baseline biometrics were retained in the model only if they improved model fit. Further adjustments were made to account for duration of T2DM, death during follow-up, length of follow-up, and financial years. Stata (version 15) was used for all analyses.

## RESULTS

### Participant characteristics

Baseline characteristics of participants with and without SMI are summarised in Table 1. A total of 2192 people with T2DM and SMI were matched to 7773 people with T2DM and no SMI; 87.7% of participants with T2DM and SMI matched to three or four controls, 53.0% of cases had schizophrenia, and 32.0% had bipolar disorder. Proportions of participants with SMI were similar to those without SMI in terms of age at time of T2DM diagnosis and at start of followup, sex, duration of T2DM, and follow-up length, but were more likely to live in areas of greatest deprivation, have dementia or learning disability, and less likely to have physical comorbidities recorded. People with SMI were also much more likely to be prescribed antidepressants and antipsychotics, and slightly less likely to be prescribed antihypertensive and lipid-lowering medications.

**Table 1.** Baseline characteristics of patients with T2DM and SMI (cases) and patients with T2DM and no SMI (controls)

		**Overall**	**Cases**	**Controls**	
**Participants^a^**		9965 (100)	2192 (22.0)	7773 (78.0)	

**Controls per case**					
4			1599 (72.9)		
3			323 (14.7)		
2			138 (6.3)		
1			132 (6.0)		

**Age at diagnosis in years, mean (SD)**					
SMI			47.98 (17.40)		
T2DM		57.83 (12.97)	56.81 (13.19)	58.12 (12.89)	

**SMI type**					
Schizophrenia			1161 (53.0)		
Schizoaffective disorder			113 (5.2)		
Bipolar disorder			701 (32.0)		
Depression and psychosis			184 (8.4)		
Other affective disorder			26 (1.2)		
Mixed			7 (0.3)		

**Age at start of follow-up in years, mean (SD)**		58.63 (12.83)	57.67 (13.11)	58.90 (12.74)	

**Duration of T2DM in years, mean (SD)**		0.82 (2.85)	0.89 (3.02)	0.80 (2.80)	

**Length of follow-up in years, mean (SD)**		6.19 (4.43)	6.02 (4.45)	6.23 (4.43)	

**Family history of diabetes**		1766 (17.7)	324 (14.8)	1442 (18.6)	

**Sex**					
Male		4758 (47.7)	1051 (47.9)	3707 (47.7)	
Female		5207 (52.3)	1141 (52.1)	4066 (52.3)	

**Ethnicity**					
White		8095 (81.2)	1826 (83.3)	6269 (80.7)	
Asian		638 (6.4)	139 (6.3)	499 (6.4)	
Black		363 (3.6)	106 (4.8)	257 (3.3)	
Mixed, other and unknown		869 (8.7)	121 (5.5)	748 (9.6)	

**Deprivation quintile using IMD 2010**					
1 (least deprivation)		1490 (15.0)	279 (12.7)	1211 (15.6)	
2		1860 (18.7)	358 (16.3)	1502 (19.3)	
3		1984 (19.9)	415 (18.9)	1569 (20.2)	
4		2287 (23.0)	542 (24.7)	1745 (22.4)	
5		2334 (23.4)	595 (27.1)	1739 (22.4)	
Data missing		10 (0.1)	3 (0.1)	7 (0.1)	

**Comorbidities**					
CVD		1591 (16.0)	285 (13.0)	1306 (16.8)	
Hypertension		4318 (43.3)	734 (33.5)	3584 (46.1)	
Dementia		64 (0.6)	32 (1.5)	32 (0.4)	
Learning disability		40 (0.4)	19 (0.9)	21 (0.3)	
CCI comorbidity, mean (SD)		0.53 (0.78)	0.49 (0.73)	0.54 (0.79)	

**Medication type**					
Antidepressants		2585 (25.9)	1062 (48.4)	1523 (19.6)	
Antipsychotics					
First generation		524 (5.3)	434 (19.8)	90 (1.2)	
Second generation		1009 (10.1)	957 (43.7)	52 (0.7)	
Antidiabetes		2164 (21.7)	521 (23.8)	1643 (21.1)	
Antihypertensives		5349 (53.7)	1000 (45.6)	4349 (56.0)	
Lipid-lowering		3361 (33.7)	684 (31.2)	2677 (34.4)	

**Smoking status**					
Non-smoker		2775 (27.8)	544 (24.8)	2231 (28.7)	
Ex-smoker		2049 (20.6)	390 (17.8)	1659 (21.3)	
Current smoker		1873 (18.8)	650 (29.7)	1223 (15.7)	
Missing data		3268 (32.8)	608 (27.7)	2660 (34.2)	

**Biometric measures**					
BMI, kg/m^2^, mean (SD)	32.66 (6.95)		32.97 (6.99)	32.56 (6.94)	
<20	62 (0.6)		12 (0.5)	50 (0.6)	
20–24	617 (6.2)		145 (6.6)	472 (6.1)	
25–29	1752 (17.6)		401 (18.3)	1351 (17.4)	
30–39	3004 (30.1)		733 (33.4)	2271 (29.2)	
≥40	871 (8.7)		221 (10.1)	650 (8.4)	
Missing data	3659 (36.7)		680 (31.0)	2979 (38.3)	
HbA1c, %, mean (SD)	7.88 (1.95)		7.82 (1.99)	7.90 (1.95)	
≤7.5^b^	2998 (30.1)		672 (30.7)	2326 (29.9)	
>7.5^b^	2188 (22.0)		460 (21.0)	1728 (22.2)	
Missing data	4779 (48.0)		1060 (48.4)	3719 (47.8)	
Total cholesterol, mmol/L, mean (SD)	5.30 (1.31)		5.36 (1.42)	5.28 (1.28)	
≤5	3477 (34.9)		722 (32.9)	2755 (35.4)	
>5	4105 (41.2)		892 (40.7)	3212 (41.3)	
Missing data	2383 (23.9)		578 (26.4)	1805 (23.2)	
Systolic BP, mmHg, mean (SD)	139.17 (18.28)		135.82 (18.16)	140.13 (18.21)	
≤140	5154 (51.7)		1280 (58.4)	3874 (49.8)	
>140	3306 (33.2)		605 (27.6)	2701 (34.7)	
Missing data	1505 (15.1)		307 (14.0)	1198 (15.4)	
Diastolic BP, mmHg, mean (SD)	81.87 (10.72)		81.47 (10.72)	81.99 (10.71)	
≤80	4391 (44.1)		1011 (46.1)	3380 (43.5)	
>80	4069 (40.8)		874 (39.9)	3195 (41.1)	
Missing data	1505 (15.1)		307 (14.0)	1198 (15.4)	

a *Data presented as* n *(%) unless otherwise stated.*

b *58 mmol/mol. BMI = body mass index. BP = blood pressure. CCI = Charlson Comorbidity Index. CVD = cardiovascular disease. IMD = Index of Multiple Deprivation. SD = standard deviation. SMI = severe mental illness. T2DM = type 2 diabetes mellitus.*

Proportions of missing values were generally similar between people with and without SMI for smoking status, biometric variables, and level of deprivation, but BMI and smoking status were slightly better recorded for people with SMI. On average, people with SMI had slightly higher BMI and levels of serum cholesterol, lower mean HbA1c, and lower mean systolic and diastolic BP.

Crude outcomes for the two participant groups are summarised in Table 2. People with SMI had, on average, a higher number of primary care consultations and received more health checks for BMI compared with those without SMI. The mean crude consultation rate was 13.65 per year for people with SMI, including 8.98 contacts with primary care physicians and 4.67 with practice nurses; for people without SMI, those rates were 11.18, 6.85, and 4.32 per year, respectively. Frequency of health checks for HbA1c and cholesterol levels were similar in both groups, whereas BP checks were fewer for people with SMI.

**Table 2.** Crude healthcare use and health outcomes of patients with T2DM and SMI (cases) and patients with T2DM and no SMI (controls)

	**Overall**	**Cases**	**Controls**
**Participants^a^**	9965 (100)	2192 (22.0)	7773 (78.0)

**Primary care consultations per year**			
Overall			
Mean (SD)	11.72 (10.05)	13.65 (10.07)	11.18 (9.97)
Median (range)	9.60 (0.00–365.30^b^)	11.10 (0.00–113.00)	9.20 (0.00–365.30^b^)
Primary care physicians			
Mean (SD)	7.32 (7.77)	8.98 (7.83)	6.85 (7.69)
Median (range)	5.70 (0.00–365.30^b^)	7.10 (0.00–91.30)	5.30 (0.00–365.30^b^)
Practice nurses			
Mean (SD)	4.40 (5.29)	4.67 (5.26)	4.32 (5.30)
Median (range)	3.30 (0.00–143.40)	3.40 (0.00–68.30)	3.20 (0.00–143.40)

**Health checks per year**			
HbA1c			
Mean (SD)	1.80 (1.16)	1.78 (1.27)	1.81 (1.13)
Median (range)	1.70 (0.00–45.70)	1.70 (0.00–28.10)	1.70 (0.00–45.70)
BP			
Mean (SD)	3.01 (4.27)	2.93 (2.49)	3.03 (4.66)
Median (range)	2.60 (0.00–365.30^b^)	2.50 (0.00–52.20)	2.60 (0.00–365.30^b^)
Total cholesterol			
Mean (SD)	1.35 (0.86)	1.38 (0.97)	1.35 (0.82)
Median (range)	1.30 (0.00–26.10)	1.30 (0.00–26.10)	1.30 (0.00–13.50)
BMI			
Mean (SD)	1.98 (4.07)	2.08 (1.95)	1.95 (4.49)
Median (range)	1.60 (0.00–365.30^b^)	1.70 (0.00–30.40)	1.60 (0.00–365.30^b^)

**Macrovascular complications — MI, PVD, and stroke — combined**	868 (8.7)	184 (8.4)	684 (8.8)

**MI**	344 (3.5)	70 (3.2)	274 (3.5)

**PVD**	305 (3.1)	58 (2.6)	247 (3.2)

**Stroke**	293 (2.9)	72 (3.3)	221 (2.8)

**Angina**	324 (3.3)	55 (2.5)	269 (3.5)

**Chronic IHD**	101 (1.0)	17 (0.8)	84 (1.1)

**Hospital admissions for CVD per year, mean (SD)**			
Emergency	0.03 (1.20)	0.03 (0.25)	0.02 (0.18)
Elective	0.01 (0.15)	0.01 (0.05)	0.01 (0.16)
Angina (ICD-10 code: I20^c^)	0.01 (0.06)	0.01 (0.06)	0.01 (0.07)
MI (ICD-10 codes: I21 and I22^c^)	0.01 (0.17)	0.01 (0.24)	0.01 (0.15)
Chronic IHD (ICD-10 code: I25^c^)	0.01 (0.15)	0.01 (0.05)	0.01 (0.17)
Stroke (ICD-10 codes: I60–I64^c^)	0.01 (0.07)	0.01 (0.08)	0.01 (0.07)

**Mortality**			
All-cause	1384 (13.9)	364 (16.6)	1020 (13.1)
CVD	511 (5.1)	132 (6.0)	379 (4.9)

a *Data presented as* n *(%) unless otherwise stated.*

b *High consultation and health-check rates reflect few consultations recorded over a short follow-up period.*

c *ICD-10 code(s) used to classify admission. BMI = body mass index. BP = blood pressure. CVD = cardiovascular disease. ICD-10 = International Classification of Diseases (10th revision). IHD = ischaemic heart disease. MI = myocardial infarction. PVD = peripheral vascular disease. SD = standard deviation. SMI = severe mental illness. T2DM = type 2 diabetes mellitus.*

For CVD, people with SMI had lower crude risks for MI, PVD, angina, chronic IHD, and macrovascular complications combined; however, the crude prevalence of stroke was higher in people with SMI than in people without SMI. Hospital-admission rates for these conditions were similar between both groups, although emergency admission rates were slightly higher in those with SMI. All-cause and CVD-specific crude mortality rates were higher in people with SMI than in those without SMI.

**Figure 1.**
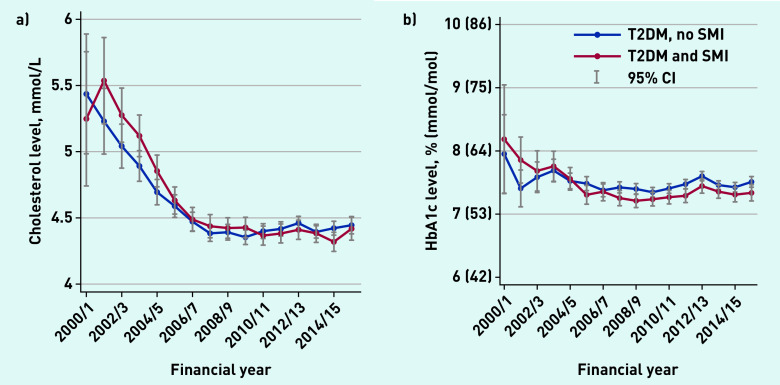
*a) Average levels of serum total cholesterol (health outcomes), 2000–2016. b) Average HbA1c levels (health outcomes), 2000–2016. HbA1c = glycated haemoglobin. SMI = severe mental illness. T2DM = type 2 diabetes mellitus.*

**Figure 2.**
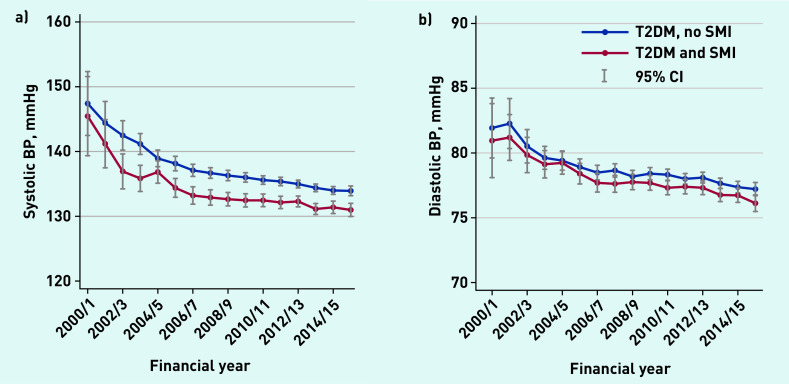
*a) Average systolic blood pressure levels (health outcomes), 2000–2016. b) Average diastolic blood pressure levels (health outcomes), 2000–2016. BP = blood pressure. SMI = severe mental illness. T2DM = type 2 diabetes mellitus.*

Average serum cholesterol levels and HbA1c levels (Figure 1) declined between 2000/01 and 2006/07 for both groups, and then remained relatively stable thereafter. Average BP levels declined throughout the study period, and people with SMI had lower levels at all time points (Figure 2).

### Regression analyses

The adjusted impact of SMI on outcomes is summarised in Table 3. Compared with participants without SMI, those with SMI had higher primary care consultation rates and were more likely to receive checks for their BP, cholesterol levels, and BMI. The estimated increase was 10% (incidence rate ratio [IRR] 1.101, 95% confidence interval [CI] = 1.069 to 1.134) for overall consultations, and 15% (IRR 1.149, 95% CI = 1.111 to 1.118) for contacts with primary care physicians. Checks were increased by 2% (IRR 1.024, 95% CI = 1.003 to 1.046) for BP, 4% (IRR 1.038, 95% CI = 1.019 to 1.058) for cholesterol, and 7% (IRR 1.068, 95% CI = 1.044 to 1.093) for BMI for people with SMI, compared with those without SMI.

**Table 3.** Adjusted impact of SMI on healthcare use and health outcomes^a^

	**Diagnosis of SMI**		

	**Adjusted IRR**	**95% CI**	***P-*value**
**Primary care consultations**			
Overall	1.101	1.069 to 1.134	<0.001
Primary care physicians	1.149	1.111 to 1.188	<0.001
Practice nurses	1.020	0.982 to 1.060	0.297
**Physical health checks**			
BP	1.024	1.003 to 1.046	0.028
Cholesterol	1.038	1.019 to 1.058	<0.001
HbA1c	0.989	0.970 to 1.009	0.297
BMI	1.068	1.044 to 1.093	<0.001

**Hospital admissions for CVD**			
Emergency	1.149	0.959 to 1.378	0.132
Angina (ICD-10 code: I20^b^)	1.532	1.069 to 2.195	0.020
MI (ICD-10 codes: I21 and I22^b^)	0.683	0.482 to 0.967	0.032
Stroke (ICD-10 codes: I60–I64^b^)	1.440	1.055 to 1.965	0.022
Elective	0.644	0.470 to 0.882	0.006
Chronic IHD (ICD-10 code: I25^b^)	0.682	0.508 to 0.915	0.011

	**Adjusted OR^b^**	**95% CI**	***P-*value**

**Primary care diagnosis of CVD**			
Macrovascular complications combined (MI, stroke, and PVD)	0.970	0.794 to 1.185	0.765
Angina	0.671	0.450 to 1.001	0.050
MI	0.929	0.698 to 1.236	0.613
Stroke	1.381	1.036 to 1.841	0.028
Chronic IHD	0.742	0.394 to 1.399	0.356

	**Adjusted HR**	**95% CI**	***P-*value**

**Mortality**			
All-cause	1.919	1.602 to 2.300	<0.001
CVD	2.242	1.547 to 3.250	<0.001

a *Models were adjusted for: age; ethnicity; level of deprivation; financial years; statistically significant confounders in comorbidities; medications use; duration and family history of T2DM; death in follow-up period; smoking status; and biometric measures.*

b *ICD-10 code(s) used to classify admissions BMI = body mass index. BP = blood pressure. CVD = cardiovascular disease. HR = hazard ratio. ICD-10 = International Classification of Diseases (10th revision). IHD = ischaemic heart disease. IRR = incidence rate ratio. MI = myocardial infarction. OR = odds ratio. PVD = peripheral vascular disease. SMI = severe mental illness. T2DM = type 2 diabetes mellitus.*

People with SMI were less likely to have a primary care diagnosis of angina (odds ratio [OR] 0.671, 95% CI = 0.450 to 1.001) but more likely to have a diagnosis of stroke (OR 1.381, 95% CI = 1.036 to 1.841). For emergency admissions, people with SMI had varied risks for different types of CVD, including increased risk for angina (IRR 1.532, 95% CI = 1.069 to 2.195) and stroke (IRR 1.440, 95% CI = 1.055 to 1.965), and decreased risk for MI (IRR 0.683, 95% CI = 0.482 to 0.967). They were less likely to have an elective admission for CVD (IRR 0.644, 95% CI = 0.470 to 0.882) and had lower admission rates for chronic IHD (IRR 0.682, 95% CI = 0.508 to 0.915) compared with people without SMI.

For people with SMI, their all-cause mortality rate was 92% higher (hazard ratio [HR] 1.919, 95% CI = 1.602 to 2.300) and their CVD-specific mortality rate was 124% higher (HR 2.242, 95% CI = 1.547 to 3.250) than that of people without SMI.

Full results of adjusted models are detailed in Supplementary Tables S1–S6. Predictors for more frequent consultations were greater deprivation, longer duration of T2DM, comorbidity, and some medications (antihypertensive, antidiabetes, antidepressants, and antipsychotics). Dementia was associated with fewer consultations. Higher frequencies of health checks were associated with lower levels of deprivation (for cholesterol and HbA1c checks), CCI comorbidities, use of medications, obesity, family history of diabetes, and higher biometric measures at baseline.

History of CVD and use of lipid-lowering medications were the strongest predictors for future CVD events, such as hospital admissions. Baseline antihypertensive prescriptions were associated with increased risk of hospital admission for angina and stroke. Longer duration of T2DM was associated with increased risk of CVD admissions; greater deprivation, history of CVD, and the presence of comorbidities were associated with increased risk of all-cause mortality.

## DISCUSSION

### Summary

After adjusting for confounders, no evidence was found that people with SMI and diabetes experienced reduced access to routine primary care, such as consultations and physical health checks, than people with diabetes alone. People with SMI, compared with those without SMI, were, however, more likely to be socioeconomically disadvantaged, more likely to have some recorded conditions (for example, dementia), and were less likely to have others (for example, physical comorbidity).

There were complex associations between SMI and the risk of CVD outcomes across diagnosis groups and healthcare settings: recorded prevalence of angina was lower for people with SMI, as were elective hospital-admission rates for CVD and emergency admission rates for MI. In contrast, emergency hospital-admission rates for angina and stroke were substantially higher. Finally, people with SMI were more likely to die compared with those without SMI, and had more than double the risk of CVD-related mortality.

### Strengths and limitations

A large, linked, longitudinal dataset of individual primary care records was analysed, allowing for multiple elements along the care pathway for diabetes and CVD to be studied. In addition, the matched, nested, case–control study, along with the design and application of within estimators reduced the impact of unobserved confounders. The interrogation of multiple diagnosis groups can be considered as sensitivity checks of the key findings, and adjusting for medication prescriptions improved the identification of comorbidities — particularly for the physical long-term conditions in people with SMI. Furthermore, as patient characteristics in the CPRD database have been shown to be broadly representative of the general UK population in terms of age, sex, and ethnicity,^22^ the findings are likely to have wide generalisability.

As with all studies that use routine healthcare records, limitations arise as a result of data accuracy and completeness of data. There were high levels of missing data for some variables and it was not possible to adjust for factors such as lifestyle, or for environmental and social determinants of health. In addition, coding behaviour is likely to vary by individual primary care staff and practices, presenting further potential errors in primary care records. Under the QOF, however, primary care providers have been incentivised to maintain registers of various conditions, including SMI; as such, the authors expect that diagnosis is less likely to be affected by this limitation compared with other areas.

Data on pathways, including referral and outpatient records, were limited; this restricted exploration of service use along the full care pathway and potential service gaps. Severity of mental illness or diabetes is not routinely recorded and could not be inferred reliably.

Heart failure, which is more common in people with diabetes compared to the general population, was not analysed; the authors did not make inference with regard to the prevalence and outcome of heart failure, and did not expect this would significantly affect findings.

Diagnosis order between SMI and T2DM is an important factor in relation to the disease development mechanism, patient demographic distribution, and potential impact on health outcomes. In this study, however, the authors did not separate those diagnosed with SMI first from those who were diagnosed with T2DM first and therefore had produced ‘aggregated’ results. More detailed analysis by diagnosis order would be helpful for future research.

As with all observational studies, it was not possible to control for unobserved confounders and systematic measurement biases that might lead to an over- or underestimatation of associations between risk factors and outcomes.

### Comparison with existing literature

The analysis of both primary care and hospital-admission records has highlighted two potential inequalities in the identification and subsequent treatment of CVD. First, compared with people without SMI, diagnosis of angina in primary care was lower in people with SMI but emergency admission rates were higher. This may reflect more rapid and severe onset, presentation, and diagnostic delay, and/or greater preference for emergency hospital services as the first point of contact for people with SMI who are experiencing chest pain. The lower admission rate for MI might also be related to this and suggests fewer, but more fatal, admissions for coronary heart disease, rather than an indicator of better outcomes. Second, the lower diagnosis and elective admission rates for IHD in people with SMI suggests that this population was less likely to be referred to cardiovascular specialist care; this finding is consistent with those of an Australian study.^26^

There are several interrelated potential explanations. Reasons for systematic underdiagnosis of CVD could include symptom under-reporting by people with SMI, diagnostic overshadowing, and a lack of confidence by mental health professionals to diagnose and manage physical comorbidities.^27^ Primary care providers might be reluctant to prescribe medications for long-term conditions and refer people with SMI for standard surgical procedures,^28^^,^^29^ perhaps due to perceived psychological stress, capacity for post-operative care, and a higher risk of developing complications after surgical interventions. A lack of integration among primary care, specialist physical healthcare, and psychiatric services may have made navigation of the care pathway more difficult for people with SMI.^30^^,^^31^

### Implications for practice

This analysis shows that the greatest challenge to the healthcare system, policymakers, and researchers who are interested in addressing the issue of health inequality in people with SM, is no longer general monitoring of metabolic risk factors as incentivised by national guidelines, as these now appear to be equally delivered to people with and without SMI. Rather, people with SMI appear to be underdiagnosed for CVD in primary care and, consequently, have poorer access to specialist and elective hospital care, leading to an elevated risk of cardiovascular mortality in this population. Policies to reduce excess deaths should, therefore, focus on activities that occur earlier in the care pathway to facilitate early diagnosis and timely treatment for CVD.

Current QOF targets do not consider the implications of antipsychotic medication and other challenges that people with SMI face in managing their diabetes.^32^ Living with greater levels of deprivation can also increase the likelihood of developing chronic health problems for people with SMI, and reduce capacity to successfully manage them. Policies should be designed to encourage primary care providers to initiate effective conversations with patients with SMI and their carers on both mental and physical health needs, improve coordination between primary care and specialist physical healthcare services, and develop strategies for tackling the particular challenges faced by people with SMI who are dealing with multimorbidity.
